# Dosimetric evaluation of Acuros XB Advanced Dose Calculation algorithm in heterogeneous media

**DOI:** 10.1186/1748-717X-6-82

**Published:** 2011-07-19

**Authors:** Antonella Fogliata, Giorgia Nicolini, Alessandro Clivio, Eugenio Vanetti, Luca Cozzi

**Affiliations:** 1 Medical Physics Unit, Oncology Institute of Southern Switzerland, Bellinzona, Switzerland

**Keywords:** dose calculation algorithm, Acuros, AAA, VMC++, inhomogeneity

## Abstract

**Background:**

A study was realised to evaluate and determine relative figures of merit of a new algorithm for photon dose calculation when applied to inhomogeneous media.

**Methods:**

The new Acuros XB algorithm implemented in the Varian Eclipse treatment planning system was compared against a Monte Carlo method (VMC++), and the Analytical Anisotropic Algorithm (AAA). The study was carried out in virtual phantoms characterized by simple geometrical structures. An insert of different material and density was included in a phantom built of skeletal-muscle and HU = 0 (setting "A"): Normal Lung (lung, 0.198 g/cm^3^); Light Lung (lung, 0.035 g/cm^3^); Bone (bone, 1.798 g/cm^3^); another phantom (setting "B") was built of adipose material and including thin layers of bone (1.85 g/cm^3^), adipose (0.92 g/cm^3^), cartilage (1.4745 g/cm^3^), air (0.0012 g/cm^3^). Investigations were performed for 6 and 15 MV photon beams, and for a large (13 × 13 cm^2^) and a small (2.8 × 13 cm^2^) field.

**Results:**

Results are provided in terms of depth dose curves, transverse profiles and Gamma analysis (3 mm/3% and 2 mm/2% distance to agreement/dose difference criteria) in planes parallel to the beam central axis; Monte Carlo simulations were assumed as reference. Acuros XB gave an average gamma agreement, with a 3 mm/3% criteria, of 100%, 86% and 100% for Normal Lung, Light Lung and Bone settings, respectively, and dose to medium calculations. The same figures were 86%, 11% and 100% for AAA, where only dose rescaled to water calculations are possible.

**Conclusions:**

In conclusion, Acuros XB algorithm provides a valid and accurate alternative to Monte Carlo calculations for heterogeneity management.

## Background

A new photon dose calculation algorithm has recently been implemented in the Eclipse treatment planning system (Varian Medical Systems, Palo Alto, USA). This algorithm, named Acuros^® ^XB Advanced Dose Calculation (Acuros XB in the following) belongs to the class of the Linear Boltzmann Transport Equation (LBTE) Solvers. LBTE solvers, similarly to those used in Monte Carlo methods, aim to allow for accurate modelling of dose deposition in media.

Many studies explored the accuracy of algorithms for photon dose calculation in materials different from water. In 2006 a classification was proposed dividing algorithms into "type a" and "type b" groups (Knöös *et *al [1]), according to management (type b) or non management (type a) of the electron transport in dose calculation. "Type b" algorithms present higher accuracy in heterogeneous media, in particular for very low density tissues [2]. The differences observed in phantom studies are partially mitigated in patients, where there is a predominance of soft tissues, more similar to water [1]; in cases with large volumes of air or low density media the differences remained largely in favour of "type b" models [3].

Many studies have also been published to compare different algorithms with Monte Carlo simulations or measurements: the Anisotropic Analytical Algorithm (AAA) was evaluated e.g. by van Esch [4], Fogliata [2], daRosa [5]. Results showed that accuracy significantly depends on energy, field size, and density of the materials. Algorithms allowing calculation of dose-to-medium lead to better agreement with Monte Carlo as already shown by Siebers [6] and confirmed by Knöös [1]. The clinical applicability of dose-to-medium calculations is limited to few systems and the new Acuros XB is included in this list. The first works on validation and evaluation of the Acuros XB algorithm were recently published by Fogliata *et al *[7] and Bush *et al *[8] showing very promising results compared to both measurements and Monte Carlo calculations.

The present report summarises a study conducted to investigate the performance and accuracy of the Acuros XB in its Eclipse implementation, when applied to materials different from water. Tests are performed in simple geometrical phantoms with inserts or layers of different materials for photon beams. Acuros XB calculations are performed using both dose-to-medium and dose-to-water options. The validation assumes, as benchmark, Voxel Monte Carlo (VMC++) simulations. To complete the comparative analysis, results are reported also for the latest version of the AAA, the "type b" algorithm currently implemented in the Eclipse TPS.

## Methods

### The algorithms

#### The Acuros XB Advanced Dose Calculation algorithm

The Acuros XB is based on the application of the LBTE that describes the interactions of radiation particles with matter. This is based on approximate numerical methods. Monte Carlo (MC) and explicit LBTE solvers, as Acuros XB, should converge to the same final results. In practice, both methods are affected by potential inaccuracies depending on the level of sampling of the probability distribution functions applied during MC simulations or to the application of variables discretisation during explicit LBTE solution. A characteristic of LBTE solvers, compared to MC simulations, is the absence of uncertainties due to statistical noise in the calculated dose.

Progenitor of Acuros is the Attila algorithm [9], developed originally for nuclear physics applications, and also investigated for external photon beam dose calculations [10,11] and brachytherapy [12]. The new Acuros algorithm, based on many of the Attila methods, was adapted for external photon dose calculations and described in Vassiliev *et al *[13]. Acuros XB is the Varian implementation in the Eclipse planning system of the original Acuros algorithm.

Acuros XB implementation consists of two main components: *i) *the photon beam source model and *ii) *the radiation transport model.

The latter includes discretisation of the spatial (), energy (*E*), and angular () variables and was firstly described by Vassiliev *et al *[13] and summarised in a previous report on Acuros XB validation in water for simple fields [14].

In brief, the dose *D*_*i *_in any grid voxel *i *is given by the following equation [13]:

where σ^e^_ED _is the macroscopic electron energy deposition cross section, ρ the material density, and Ψ^*e *^the angular electron fluence. Acuros XB calculates the energy dependent electron fluence, based on the material properties of the patient, as derived from the Hounsfield Unit (HU) of the CT dataset.

Dose to medium or dose to water can be selected in Acuros XB.

When *dose to medium *is calculated, σ_ED_^e ^and ρ and are based on the material properties of output grid voxel, *i*.

When *dose to water *is reported, σ_ED_^e ^and ρ are based on water in a post processing step (the transport calculation is identical for both *dose to medium *and *dose to water *reporting); in materials different from water, the dose is defined as the dose absorbed by a volume of water which is small enough not to perturb the energy dependent electron fluence. This volume should be much smaller than the output dose grid voxel of the computer based calculation or of any detector used to measure dose to water.

The macroscopic cross sections σ used by Acuros XB are modelled as the product of two components: the microscopic cross section for a given interaction , and the mass density of the material ρ with the relationship:

where *M *is the atomic mass and *N*_*α *_is the Avogadro's number. Coupled photon-electron cross sections include Compton scatter, photo-electric effect, and pair production, but not the Rayleigh scatter. In the model, the energy from bremsstrahlung photons produced by electron interactions inside the patients is not considered, being judged not significant for energies typical in the radiotherapy range.

The cutoff for electron energy is set at 500 keV (200 keV in version 11) kinetic energy only (without rest mass) and it is not modifiable by the user.

In clinical cases, radiation transport is performed for materials derived by anatomical information: tissue segmentation is based on density ranges related to HU values read in the patient CT dataset. Density to human tissues correspondence is reported in Table 1 (for both Acuros XB versions 10 and 11); for each material the specific chemical elemental composition is based on the ICRP Report 23 [15]. In Eclipse the user can also manually assign materials (or human tissues) with predefined HU values. Acuros XB does not allow calculations for mass densities higher than 3.0 g/cm^3 ^to prevent incorrect material assignment to densities larger than the expected scale for human bones. If the CT dataset contains HU values higher than this upper limit, ad-hoc structures are defined with manual assignment of materials.

**Table 1:** Material mass densities for automatic conversion, as implemented in the two Acuros XB versions.

Material	Density Range [g/cm^3^] Acuros XB version 10	Density Range [g/cm^3^] Acuros XB version 11
Air	-	0.000-0.020
Lung	0.000-0.590	0.011-0.624
Adipose Tissue	0.590-0.985	0.554-1.001
Muscle, Skeletal	0.985-1.075	0.969-1.093
Cartilage	1.075-1.475	1.056-1.600
Bone	1.475-3.000	1.100-3.000

#### The Anisotropic Analytical Algorithm, AAA

Comparison to the AAA algorithms was also included in the study. AAA, based on the work of Ulmer *et *al [16-18] and Tillikainen *et *al [19,20], was extensively validated [2,4,21-26]. The reader should refer to Tillikainen *et *al [20] for detailed description. AAA is not accounting for chemical material/tissue properties, hence the computed dose can be defined as dose to water, rescaled according to the specific density (dose rescaled to water in the following).

#### The Voxel Monte Carlo, VMC++

The Voxel Monte Carlo VMC++ [27-30] is a class II condensed history Monte Carlo simulation of coupled electron-photon transport. It uses small angle approximation, and re-uses electron histories and STOPS (Simultaneous Transport Of Particle Sets) variance reduction techniques [31]. It was validated in the field of radiotherapy by Gardner *et *al [32].

The version of VMC++ used here is implemented as a research version in Eclipse. Material chemical composition and related density ranges are here set identical to the Acuros XB settings. For the simulations, the electron energy cutoff is automatically selected and based upon the density of the material density; a smoothing process is activated during calculations (locally adaptive Savitzky-Golay filter); final dose calculation accuracy is set to 1%. A cross validation of VMC++ version is here presented against EGSnrc as already published [2]. During EGSnrc simulations 75 million particles are used to have a maximum statistical uncertainty of about 2%. The resolution is 2.5 mm in all directions. The total energy cut-off for electrons and photons are set to 700 keV and 10 keV, respectively.

#### Source model

The source model used for this study is the standard multiple source implemented in Eclipse and is the same for all algorithms used: Acuros XB, AAA and VMC++. For a detailed description the reader can refer to Tillikainen *et *al [19].

### Eclipse framework and tested versions

All calculations are performed using the Eclipse planning system, with version 10 for Acuros XB and AAA, and version 8 for VMC++. The algorithm versions used are as following:

• Acuros XB: clinical release 10.0.28.

• AAA: clinical release 10.0.25.

• VMC++: research release 8.0.1, not for clinical usage.

Some results are also reported for the Acuros XB calculations in its engineering pre-clinical version 11.0.03. Two are the main differences between the two Acuros XB versions 10 and 11. The first concerns the human material assignment, where the Air material is assigned to very low density regions in the body (Air material is not present in version 10), and the density ranges for each material are slightly overlapping (Table 1). The second improvement refers to a better re-sampling process of the structure voxels to the calculation grid, setting the density and material of the structure to the calculation voxel when at least half of the calculation voxel volume belongs to the structure.

All calculations, are performed with a grid size of 1.25 mm. The grid, in addition to the smoothing process used in the VMC++ calculations might lead to some unavoidable smoother dose profiles.

### The phantoms and the beams

All studies are performed on a set of virtual phantoms.

**Figure 1:**
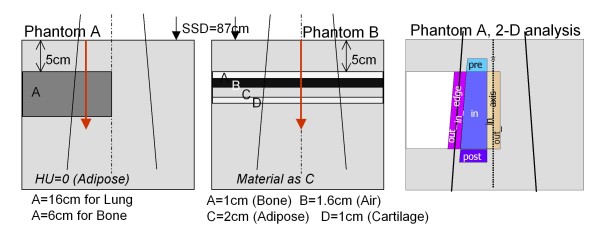
**Geometrical layout of the phantoms**. Phantom A on the left; phantom B in the middle; sectors used in the 2D gamma analysis for phantom A on the right.

Figure 1 shows a schematic representation of the phantoms which are characterized as follows [2]:

Phantom A) An insert, covering laterally only half of the entire phantom and positioned at 5 cm depth in a large phantom of HU = 0 ('Muscle Skeletal' as automatic assignment) is simulated for three different materials and thicknesses:

- *Normal Lung*: 0.198 g/cm^3^, HU = -780, lung tissue, 16 cm thick.

- *Light Lung*: 0.035 g/cm^3^, HU = -942, lung tissue, 16 cm thick.

- *Bone*: 1.798 g/cm^3^, HU = 1380, bone tissue, 6 cm thick.

Phantom B) Four thin layers of different materials (*Heter1*), starting at 5 cm depth are included in a large phantom of the same density and composition as insert C (Adipose):

- Layer A: 1.4751 g/cm^3^, HU = 763, bone tissue, 1 cm thick

- Layer B: 0.0012 g/cm^3^, HU = -993, air, 1.6 cm thick

- Layer C: 0.92 g/cm^3^, HU = -122, adipose tissue, 2 cm thick

- Layer D: 1.4745 g/cm^3^, HU = 762, cartilage tissue, 1 cm thick.

Notice that layers A and D differ for only one HU, but have different material assignment (bone or cartilage), presenting different elemental composition, especially in terms of Calcium content.

Source to phantom distance SSD is set to 87 cm, gantry and collimator to 0 degree. Doses are normalised to 3 cm depth on the beam central axis. For all phantoms calculations are performed for the following settings:

- field sizes: 2.8 × 13 cm^2^, small field, SF, (the long axis crossed the heterogeneity boundary), and 13 × 13 cm^2^, large field, LF.

- beam energies: 6 and 15 MV from a Varian Clinac 2100 iX, presenting TPR_20/10 _of 0.672 and 0.761 respectively (6X and 15X in the following).

For all cases, calculations are performed for Acuros XB and VMC++ as: *i) *dose to water, *ii) *dose to medium and *iii) *dose rescaled to water. This last modality is defined with a manual assignment to water material for all phantom structures, outline and inserts, with specific HU according to each phantom setting; CT ranges to corresponding materials and compositions are modified accordingly also for VMC++ calculations. For AAA, only the dose rescaled to water option is available.

### The analysis

#### 1-D analysis: DD and profiles

Data are reported for calculations along the directions shown by the arrows in figure 1, i.e. depth dose curves (DD) at -4 cm off-axis parallel to the beam central axis for phantom A, and on the beam central axis for phantom B.

Horizontal transverse profiles are calculated at the depth of mid-thickness of the inhomogeneities for phantom A to evaluate the lateral interface.

#### 2-D analysis: Gamma evaluation

2-D dose distributions in the vertical transversal plane through the isocentre, crossing the longest field jaw setting are evaluated. Gamma of Low analysis [33] is performed, using different threshold criteria: distance to agreement DTA = 2 mm and 3 mm, dose difference ΔD = 2%, 3%; all calculations are performed as global gamma indexes, i.e. relative to the dose at 3 cm depth on the beam central axis. VMC++ calculations are assumed as reference. Each planar dose from phantom A is divided into various sectors as depicted in figure 1:

- *pre: *before the inhomogeneity, from 3 cm depth, with 1.5 cm internal margin from the field edge on the left and the beam central axis on the right

- *in*: inside the inhomogeneity, with the same lateral margins of 1.5 cm

- *post*: after the inhomogeneity for a depth of 2 cm.

- *edge*: along the inhomogeneity, across the field edge, 1.5 cm inside and 1.5 cm outside the border

- *edge_in*: the *edge *sector only inside the field

- *edge_out*: the *edge *sector only outside the field

- *axis*: across the beam central axis (and also inhomogeneity), 1.5 cm inside and 1.5 cm outside the inhomogeneity

- *axis_in*: the *axis *sector only inside the inhomogeneity

- *axis_out*: the *axis *sector only outside the inhomogeneity

Gamma evaluation is recorded as Gamma Agreement Index, GAI, defined as the percentage of the pixels fulfilling the criteria inside each sector.

For phantom B the following regions, included in the field, are analysed:

- *pre*: before the first inhomogeneity, from 3 to 5 cm depth

- *bone*: inside the bone layer of 1 cm

- *air*: inside the air layer of 1.6 cm

- *adipose*: inside the adipose layer of 2 cm

- *cartilage*: inside the cartilage layer of 1 cm

- *post*: after the last inhomogeneity layer, for 2 cm depth.

## Results and Discussion

### Dose to medium, dose to water, dose rescaled to water

**Figure 2:**
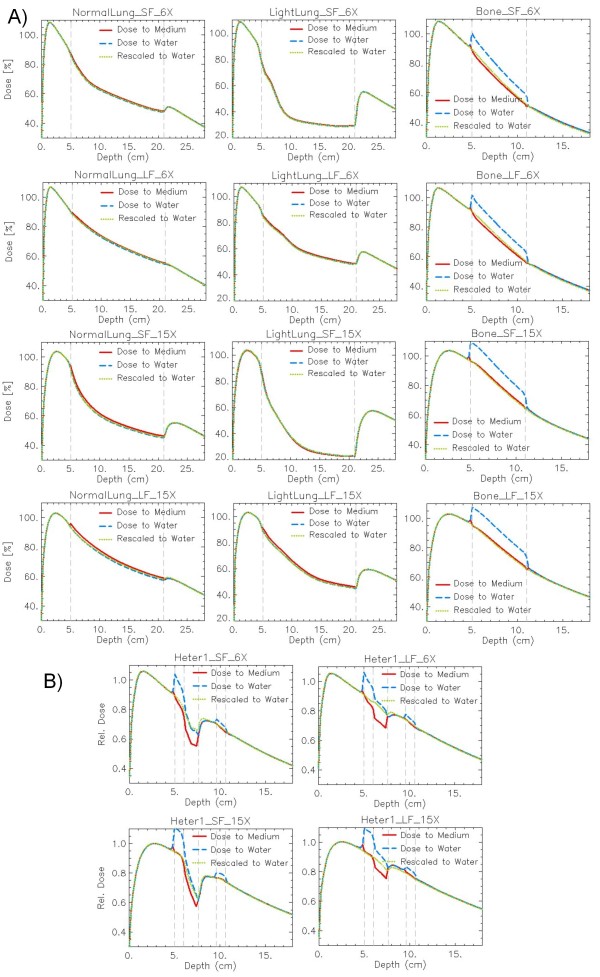
**Depth dose curves (DD) as *dose to medium*, dose to water, dose rescaled to water**. Calculations with Acuros XB version 10 algorithm: (A) Phantom A: in columns: Normal Lung, Light Lung, Bone; in rows: SF and LF for 6X, SF and LF for 15X. (B) Phantom B: in columns: SF, LF; in rows: 6X, 15X.

A summary of the DD calculated with Acuros XB as dose to medium, dose to water, and dose rescaled to water is reported in figure 2(A) for all phantom A, and in figure 2(B) for all phantom B settings. Similar results are found for VMC++ with the three calculation modalities.

The Lung cases present very small differences among all calculation modalities. In the Bone case, dose to water calculations in bone show strong difference in DD compared to the other two calculations. Dose to medium is expressed as dose to water multiplied by the stopping power ratio *s*_*water,medium *_between the two media; *s*_*water,bone *_is in the range 1.09-1.15 for cortical bone [6]. This confirms the difference of ~10-14% reported here. From a qualitative analysis of the Bone DD, a small peak about 2-3 mm before and behind the insert is computed in the dose to medium with Acuros XB. Small peaks in the dose to medium calculations are consistently present also in the horizontal profiles at the level of the interface between the two media.

Phantom B data show similar patterns depending on the layer material, with enhanced criticalities due to the short distance between interfaces and to the presence of different adjacent materials with very different density and composition, e.g. bone and air, where the different exit dose from bone is reflected in higher dose inaccuracy in the next air layer.

### VMC++ vs. EGSnrc comparison

**Figure 3:**
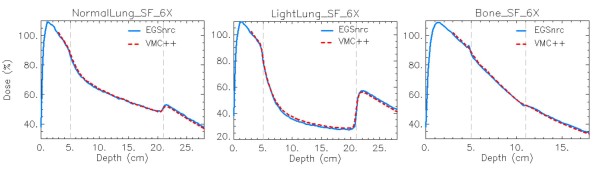
**EGSnrc and VMC++ comparison**. Depth dose curves (DD) at -4 cm off-axis for the SF, 6X case in Normal Lung, Light Lung and Bone for EGSnrc and VMC++.

Due to the non-validated nature of the used VMC++, in figure 3 a comparison between VMC++ and EGSnrc simulations for DD curves in SF and 6X cases of phantom A is presented, showing small differences between the two calculations.

### One-dimensional analysis: DD and profiles

In the following, only graphs relative to dose to medium calculations for Acuros XB, AAA and VMC++ are presented. Graphs referring to dose to water and dose rescaled to water are reported as additional files.

**Figure 4:**
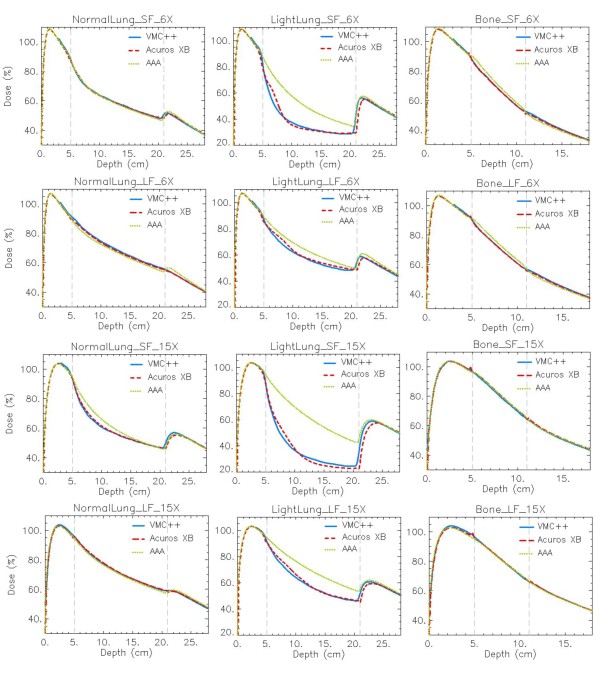
**Depth dose curves (DD) at -4 cm off-axis**. *Dose to medium *calculations for VMC++, Acuros XB version 10, and AAA in phantom A. In columns: Normal Lung, Light Lung, Bone; in rows: SF and LF for 6X, SF and LF for 15X.

**Figure 5:**
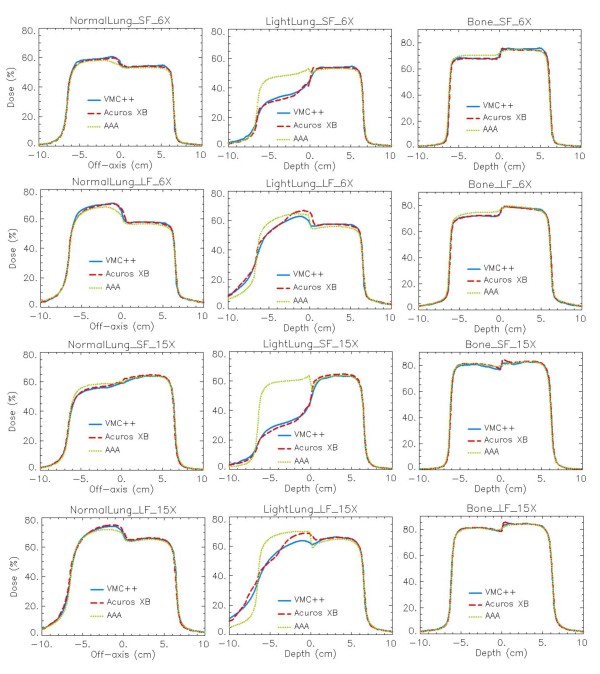
**Profiles at mid-depth of the heterogeneity insert**. *Dose to medium *calculations for VMC++, Acuros XB version 10, and AAA in phantom A. In columns: Normal Lung, Light Lung, Bone; in rows: SF and LF for 6X, SF and LF for 15X.

**Figure 6:**
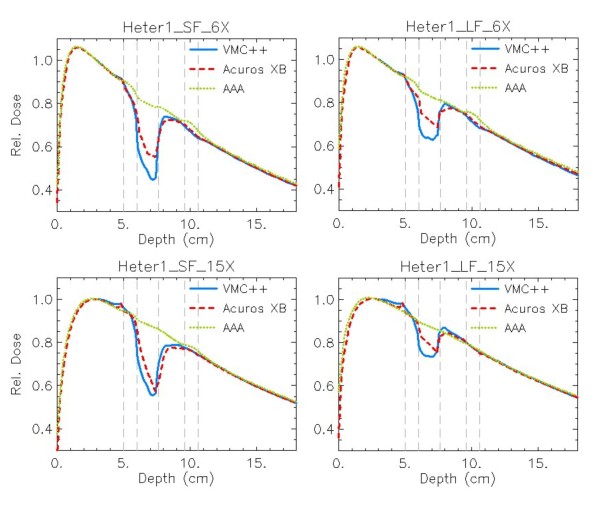
**Depth dose curves (DD) at beam central axis**. *Dose to medium *calculations for VMC++, Acuros XB version 10, and AAA in phantom B. In columns: SF, LF; in rows: 6X, 15X.

For phantom A figure 4 reports the DD curves, while figure 5 shows the horizontal profiles at mid-depth of the insert. Figure 6 presents the DD curves for phantom B. The corresponding additional figures are: Additional file 1, Figure S1 (DD, dose to water), Additional file 2, Figure S2 (DD, dose rescaled to water), Additional file 3, Figure S3 (profiles, dose to water), Additional file 4, Figure S4 (profiles, dose rescaled to water), Additional file 5, Figure S5 (DD, dose to water in phantom B), Additional file 6, Figure S6 (DD, dose rescaled to water in phantom B). To appraise the improvement of Acuros XB compared to previous analytical algorithms, the AAA calculations are always reported although Acuros XB computes transport and dose deposition in the actual material, while in AAA the transport and dose deposition uses radiological and density scaling methods. A genuine comparison for AAA calculations is provided in figures ADD-b referring to dose rescaled to water, where explicit different elemental composition of materials is not considered, and the differences mainly due to the algorithms as radiation transport models are shown.

For all calculations performed in Normal Lung tissue, good agreement between Acuros XB and VMC++ is achieved. AAA, as expected from the radiation transport model, is less accurate especially for small fields and high energy beams [2]. The rebuildup curve behind the low density insert starts at the interface layer in Acuros XB calculations, while in VMC++ computations it starts about 1 mm inside the lung insert. This effect, more evident for the Light Lung cases, yields to a shift of about 2 mm of the rebuildup portion of the curve for the two algorithms. This difference could partly ascribed to the boundary handling from different algorithms (considering that no grid alignment is performed between image and dose grid voxels), or also to the variance reduction techniques implemented in VMC++ to decrease statistical noise.

The Light Lung DD curve has a noticeably steeper gradient that starts 2-4 cm distal to the interface. The horizontal profiles through the light lung insert enhance the display of this unexpected increase in dose a few cm from the field edge and the interface, an effect that is more pronounced at deeper distances. Inside the most internal light lung material the differences between Acuros XB and VMC++ are small. The calculations for very low densities prove to be critical for all algorithms since this density range enhanced the inaccuracies coming from different approximations, as e.g. the energy cutoff for electron interactions, present also in Monte Carlo simulations.

Acuros XB and VMC++ show good mutual agreement in the bone tissue, while AAA presents inferior accuracy for low energy. The small peaks, few mm before and after the bone insert, are more pronounced for Acuros XB calculations. In dose to water calculations, Acuros XB shows the start of the increase of the depth dose curve for dose to water ~5 mm before the bone interface, while VMC++ anticipates this to ~10 mm before it.

Results from phantom B present the same, but enhanced, patterns and characteristics as phantom A. To note is the inability of AAA to properly model the presence of thin inhomogeneities.

**Figure 7:**
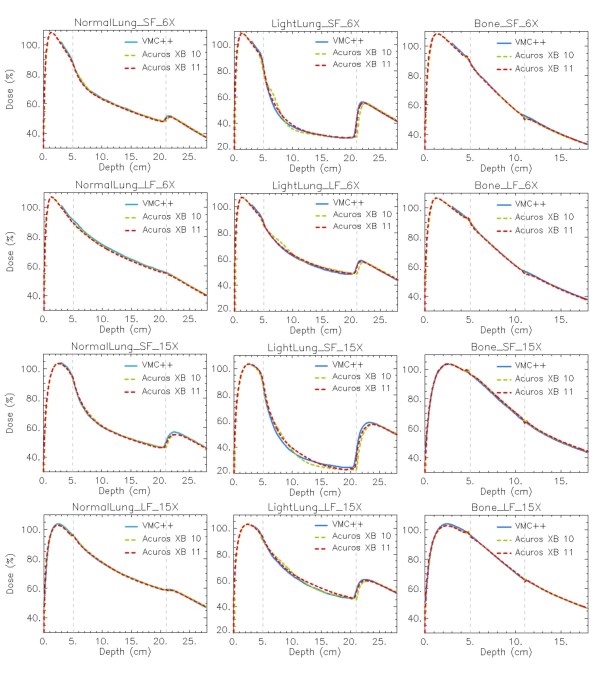
**Depth dose curves (DD) at -4 cm off-axis**. *Dose to medium *calculations for VMC++, Acuros XB versions 10 and 11 in phantom A. In columns: Normal Lung, Light Lung, Bone; in rows: SF and LF for 6X, SF and LF for 15X.

**Figure 8:**
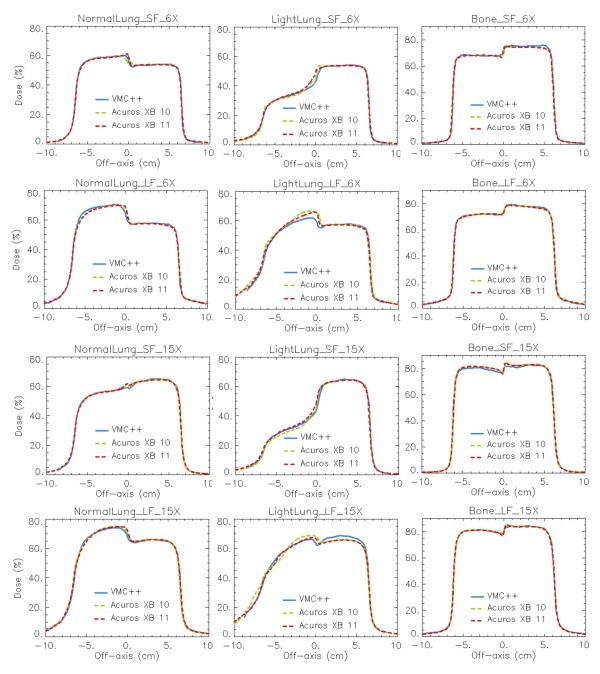
**Profiles at mid-depth of the heterogeneity insert**. *Dose to medium *calculations for VMC++, Acuros XB versions 10 and 11 in phantom A. In columns: Normal Lung, Light Lung, Bone; in rows: SF and LF for 6X, SF and LF for 15X.

**Figure 9:**
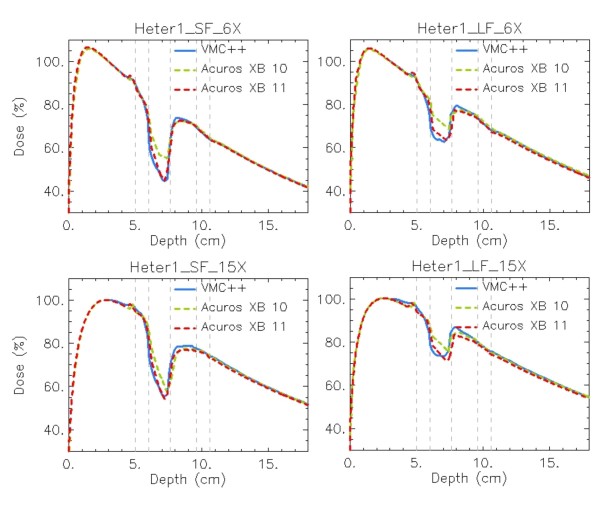
**Depth dose curves (DD) at beam central axis**. *Dose to medium *calculations for VMC++, Acuros XB versions 10 and 11 in phantom B. In columns: SF, LF; in rows: 6X, 15X.

In figures 7, 8 and 9 the same plots as in figures 4, 5 and 6 show the calculation difference between the two Acuros XB versions, benchmarked to VMC++. With the engineering pre-clinical version 11, the rebuildup after the lung insert, and the interfaces in the horizontal profiles are more accurately modelled due to the better re-sampling of the structure voxel to the calculation voxel. Also the unexpected dose patterns inside light lung insert visible for Acuros XB version 10 tend to disappear if version 11 is used.

For phantom B settings, that present thin inhomogeneity layers, a plain improvement is shown with Acuros XB version 11, due to the improved alignment of structures and dose voxels. In addition it can be noticed that the dose computed with Acuros XB version 11 inside the Air material layer presents much better agreement with VMC++ calculations, due to the inclusion in the human tissues list of the air material, that was considered as lung composition in version 10.

### Two-dimensional analysis: Gamma evaluation

**Figure 10:**
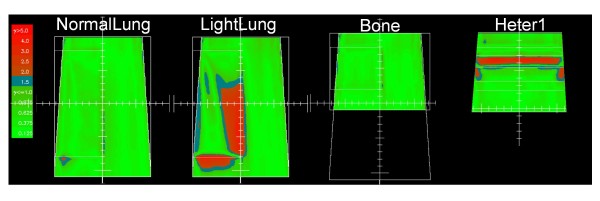
**Gamma maps**. Examples for LF, 15X, *dose to medium*, Acuros XB version 10 vs. VMC++. Thresholds 2 mm, 2% as global gamma computations. White lines represent the heterogeneity interfaces.

The main limit of any 2D analysis based on Gamma evaluation is its threshold effect, hence results have to be considered together with the dose profiles shown in the previous figures. Examples of the pass/fail patterns in the 2D planes analysed with Gamma evaluation, are shown in figure 10 for Acuros XB, dose to medium calculations, with a global gamma criteria of 2%, 2 mm. Two-dimensional analysis is here reported only for version 10 of Acuros XB, being the clinical released version at the present stage.

**Figure 11:**
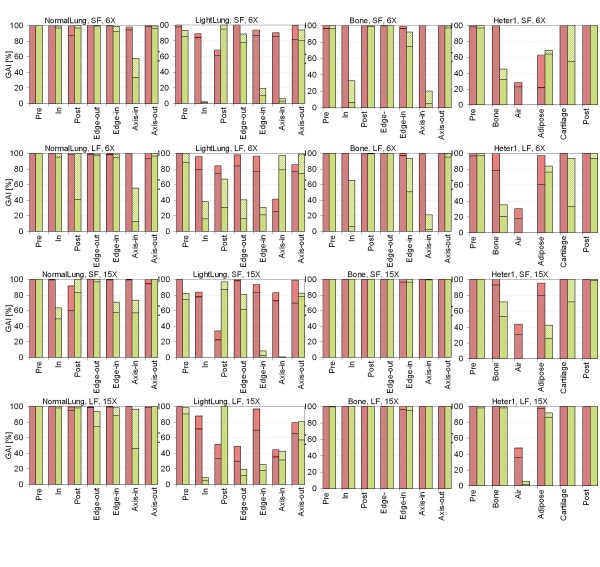
**Histograms of the GAI**. Global gamma calculation for each sector of phantoms A and B, for *dose to medium *calculations for Acuros XB version 10 (red horizontal hatching) and AAA (green diagonal hatching). Each bin represents the two threshold results of 2%, 2 mm (thin cross-hatching) and 3%, 3 mm (thick cross-hatching). In columns: Normal Lung, Light Lung, Bone, phantom B; in rows: SF and LF for 6X, SF and LF for 15X.

Figure 11 shows the summary of GAI (for global gamma calculation) for each phantom sector, both field sizes, both energies. Each bin represents the two threshold results of 2%, 2 mm (thin cross-hatching) and 3%, 3 mm (thick cross-hatching). For each sector Acuros XB (version 10), as well as AAA were analysed against VMC++ calculations. The Additional file 7, Figure S7 and the Additional File 8, Figure S8 show all cases for dose to water and dose rescaled to water, respectively. Table 2 summarises values of Gamma Agreement Index for 3%, 3 mm thresholds in all calculation modalities for phantom A setting. Data are relative to the entire insert area crossed by the beam in the plane parallel to its central axis and passing through the isocentre.

**Table 2:** Gamma Agreement Index GAI

		6X		15X	
		
		LF	SF	LF	SF
***Dose to medium***					
*NormalLung*	Acuros XB	99.9	99.6	100.0	100.0
	AAA	91.2	91.6	98.9	66.6
*LightLung*	Acuros XB	85.6	90.1	81.5	85.5
	AAA	47.9	6.3	18.4	1.6
*Bone*	Acuros XB	99.9	99.8	100.0	100.0
	AAA	62.2	41.8	100.0	100.0

***Dose to water***					
*NormalLung*	Acuros XB	99.8	99.2	100.0	99.3
	AAA	97.1	95.8	98.0	48.2
*LightLung*	Acuros XB	83.5	90.5	83.8	90.0
	AAA	34.8	6.1	12.2	1.6
*Bone*	Acuros XB	89.0	75.8	90.5	72.7
	AAA	79.8	97.4	18.3	12.6

***Dose rescaled to water***					
*NormalLung*	Acuros XB	99.9	98.7	99.9	99.3
	AAA	99.5	93.3	97.6	51.8
*LightLung*	Acuros XB	81.0	88.6	78.6	85.6
	AAA	29.9	6.0	7.8	1.6
*Bone*	Acuros XB	99.9	99.8	99.9	99.9
	AAA	100.0	100.0	100.0	100.0

3%, 3 mm thresholds in the area of the insert included in the beam in the plane parallel to the beam central axis passing through the isocentre. Benchmark dose: VMC++, test dose: AAA or Acuros XB version 10.

LF = large field, SF = small field

Summarising the results from phantom A: the GAI (3%, 3 mm criteria) for Acuros XB (version 10), dose to medium, are in average 100%, 86%, 100%, for Normal Lung, Light Lung and Bone cases respectively. The same figures are 87%, 19%, 76% for AAA calculations. Considering the dose rescaled to water, where the comparison with AAA comparison is more relevant, GAI results are: 99%, 83%, 100% for Acuros XB, and 86%, 11%, 100% for AAA.

Those data imply that for low density materials, more than the specific modality to compute dose, the critical variable is identified in the mass density of the medium itself, while the crucial point for bone tissue is more related to the elemental composition and the ability to consider it in calculations.

From phantom B results the dose inside the Air layer presents rather low gamma values for both AAA and Acuros XB version 10.

The results here presented are in good agreement with what has been published by Bush *et al *[8] comparing Acuros XB calculations with BEAMnrc/DOSXYZnrc Monte Carlo simulations. The key point from the two studies remains the high level of accuracy of Acuros XB implementation in Eclipse when simple heterogeneities in phantom are involved. Anyway, different settings have been used in the two studies, mainly in the two Monte Carlo algorithms.

In their work, Bush *et al*, used different elemental compositions and density ranges for HU to mass density conversion with respect to what is implemented in Acuros XB. This discrepancy is not used in the present paper, where the same Acuros XB chemical composition and density range are set for VMC++ calculations.

Also the electron energy cutoff is different for all calculations: 700 keV as kinetic+electron rest mass in Monte Carlo calculation from Bush *et al*; in VMC++ of the present study it is automatically selected and based upon the density of the material density; it is set to 500 keV (version 10) or 200 keV (version 11) as kinetic energy only for Acuros XB calculations.

Those two examples of differences point to unavoidable approximations of all dose calculations, including Monte Carlo. Those examples enforce the need of publishing different comparisons, presenting various characteristics, in order to give to the community the opportunity to read about results coming from different approaches.

## Conclusions

The new Acuros XB photon dose calculation engine is tested for accuracy against Monte Carlo simulations in phantoms with simple geometrical heterogeneities in its clinical version 10. The comparison is extended also to the widely used AAA algorithm. Good agreement between Acuros XB and Monte Carlo is shown, even in extreme cases of materials of very low density and for low energy and small fields. Some differences between different algorithms are pointed out at interfaces between different materials. In those cases, Acuros XB and VMC++ present differences mainly in the rebuildup region. The agreement in this region improves with the newer version 11 of the Acuros XB algorithm.

In general, results suggest that the Acuros XB algorithm is mature for clinical implementation and can provide a valid and accurate alternative to Monte Carlo calculations.

## Competing interests

The present work was partially supported by a Grant from Varian Medical Systems, Palo Alto, CA, USA.

Dr. L. Cozzi acts as Scientific Advisor to Varian Medical Systems and is Head of Research and Technological Development to Oncology Institute of Southern Switzerland, IOSI, Bellinzona.

## Authors' contributions

AF and LC coordinated the entire study. Data acquisition and 1-D analysis were conducted by AF, GN and LC. 2-D analysis was done by AC and EV. The manuscript was prepared by AF. All authors read and approved the final manuscript.

## Supplementary Material

Additional file 1 **DD in water for phantom A**. Depth dose curves (DD) at -4 cm off-axis. Dose to water calculations for VMC++, Acuros XB version 10, and AAA in phantom A. In columns: Normal Lung, Light Lung, Bone; in rows: SF and LF for 6X, SF and LF for 15X. Click here for file

Additional file 2 **DD rescaled to water for phantom A**. Depth dose curves (DD) at -4 cm off-axis. Dose rescaled to water calculations for VMC++, Acuros XB version 10, and AAA in phantom A. In columns: Normal Lung, Light Lung, Bone; in rows: SF and LF for 6X, SF and LF for 15X. Click here for file

Additional file 3 **Dose profiles to water for phantom A**. Profiles at mid-depth of the heterogeneity insert. Dose to water calculations for VMC++, Acuros XB version 10, and AAA in phantom A. In columns: Normal Lung, Light Lung, Bone; in rows: SF and LF for 6X, SF and LF for 15X. Click here for file

Additional file 4 **Dose profiles rescaled to water for phantom A**. Profiles at mid-depth of the heterogeneity insert. Dose rescaled to water calculations for VMC++, Acuros XB version 10, and AAA in phantom A. In columns: Normal Lung, Light Lung, Bone; in rows: SF and LF for 6X, SF and LF for 15X. Click here for file

Additional file 5 **DD to water for phantom B**. Depth dose curves (DD) at beam central axis. Dose to water calculations for VMC++, Acuros XB version 10, and AAA in phantom B. In columns: SF, LF; in rows: 6X, 15X. Click here for file

Additional file 6 **DD rescaled to water for phantom B**. Depth dose curves (DD) at beam central axis. Dose rescaled to water calculations for VMC++, Acuros XB version 10, and AAA in phantom B. In columns: SF, LF; in rows: 6X, 15X. Click here for file

Additional file 7 **GAI for dose to water**. Histograms of the GAI. Global gamma calculation for each sector of phantoms A and B, for dose to water calculations for Acuros XB version 10 (red horizontal hatching) and AAA (green diagonal hatching). Each bin represents the two threshold results of 2%, 2 mm (thin cross-hatching) and 3%, 3 mm (thick cross-hatching). In columns: Normal Lung, Light Lung, Bone, phantom B; in rows: SF and LF for 6X, SF and LF for 15X. Click here for file

Additional file 8 **GAI for dose rescaled to water**. Histograms of the GAI. Global gamma calculation for each sector of phantoms A and B, for dose rescaled to water calculations for Acuros XB version 10 (red horizontal hatching) and AAA (green diagonal hatching). Each bin represents the two threshold results of 2%, 2 mm (thin cross-hatching) and 3%, 3 mm (thick cross-hatching). In columns: Normal Lung, Light Lung, Bone, phantom B; in rows: SF and LF for 6X, SF and LF for 15X. Click here for file
