# Supplementary figures and images for: Dosimetric evaluation of Acuros XB Advanced Dose Calculation algorithm in heterogeneous media
Source: Radiat Oncol. 2011 Jul 19;6:82. doi: 10.1186/1748-717X-6-82 (PMC3168411; doi:10.1186/1748-717X-6-82)

## Slide 1
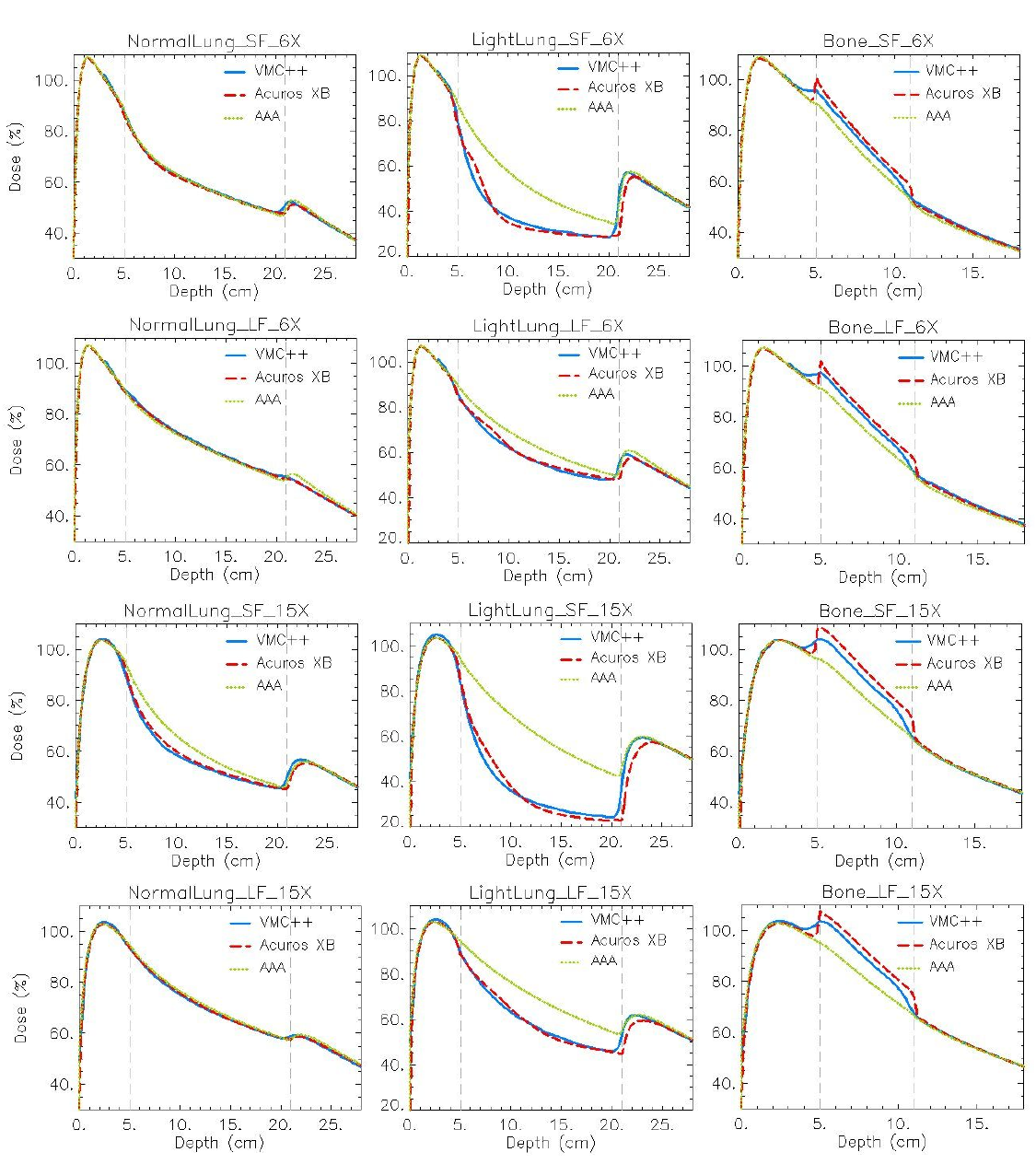

Supplement: Additional file 1 — DD in water for phantom A. Depth dose curves (DD) at -4 cm off-axis. Dose to water calculations for VMC++, Acuros XB version 10, and AAA in phantom A. In columns: Normal Lung, Light Lung, Bone; in rows: SF and LF for 6X, SF and LF for 15X. [file 1748-717X-6-82-S1.PPT]

## Slide 1
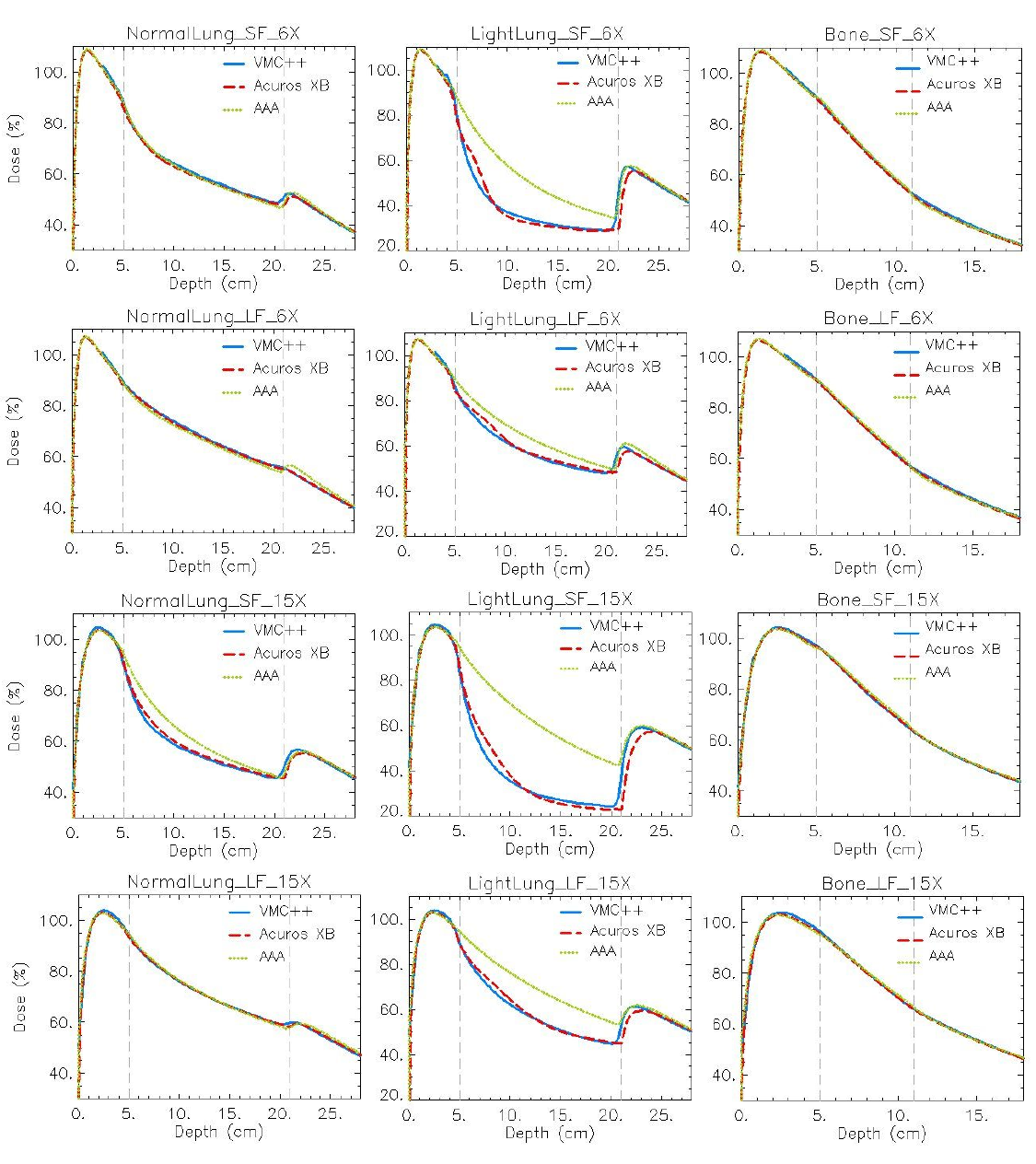

Supplement: Additional file 2 — DD rescaled to water for phantom A. Depth dose curves (DD) at -4 cm off-axis. Dose rescaled to water calculations for VMC++, Acuros XB version 10, and AAA in phantom A. In columns: Normal Lung, Light Lung, Bone; in rows: SF and LF for 6X, SF and LF for 15X. [file 1748-717X-6-82-S2.PPT]

## Slide 1
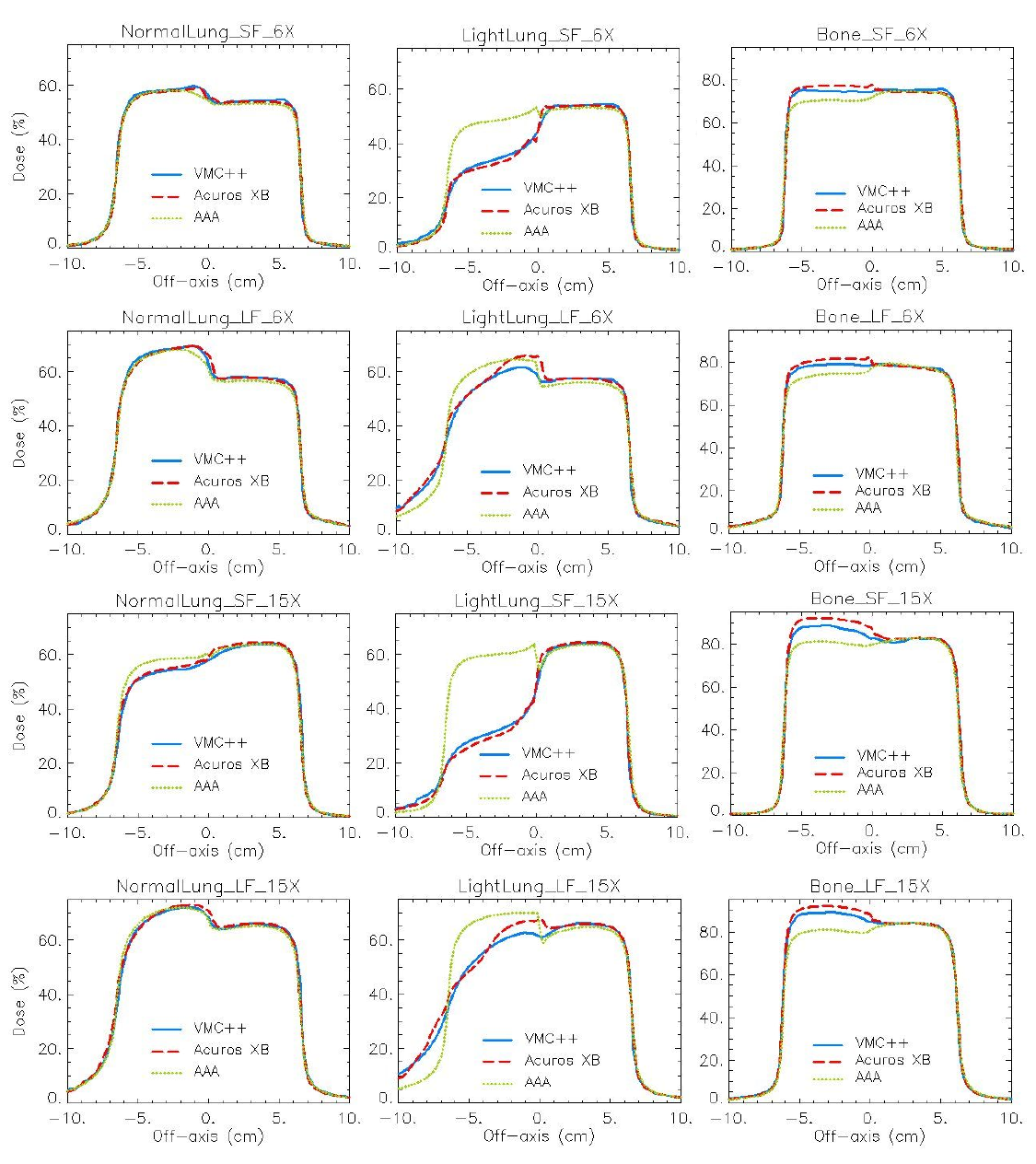

Supplement: Additional file 3 — Dose profiles to water for phantom A. Profiles at mid-depth of the heterogeneity insert. Dose to water calculations for VMC++, Acuros XB version 10, and AAA in phantom A. In columns: Normal Lung, Light Lung, Bone; in rows: SF and LF for 6X, SF and LF for 15X. [file 1748-717X-6-82-S3.PPT]

## Slide 1
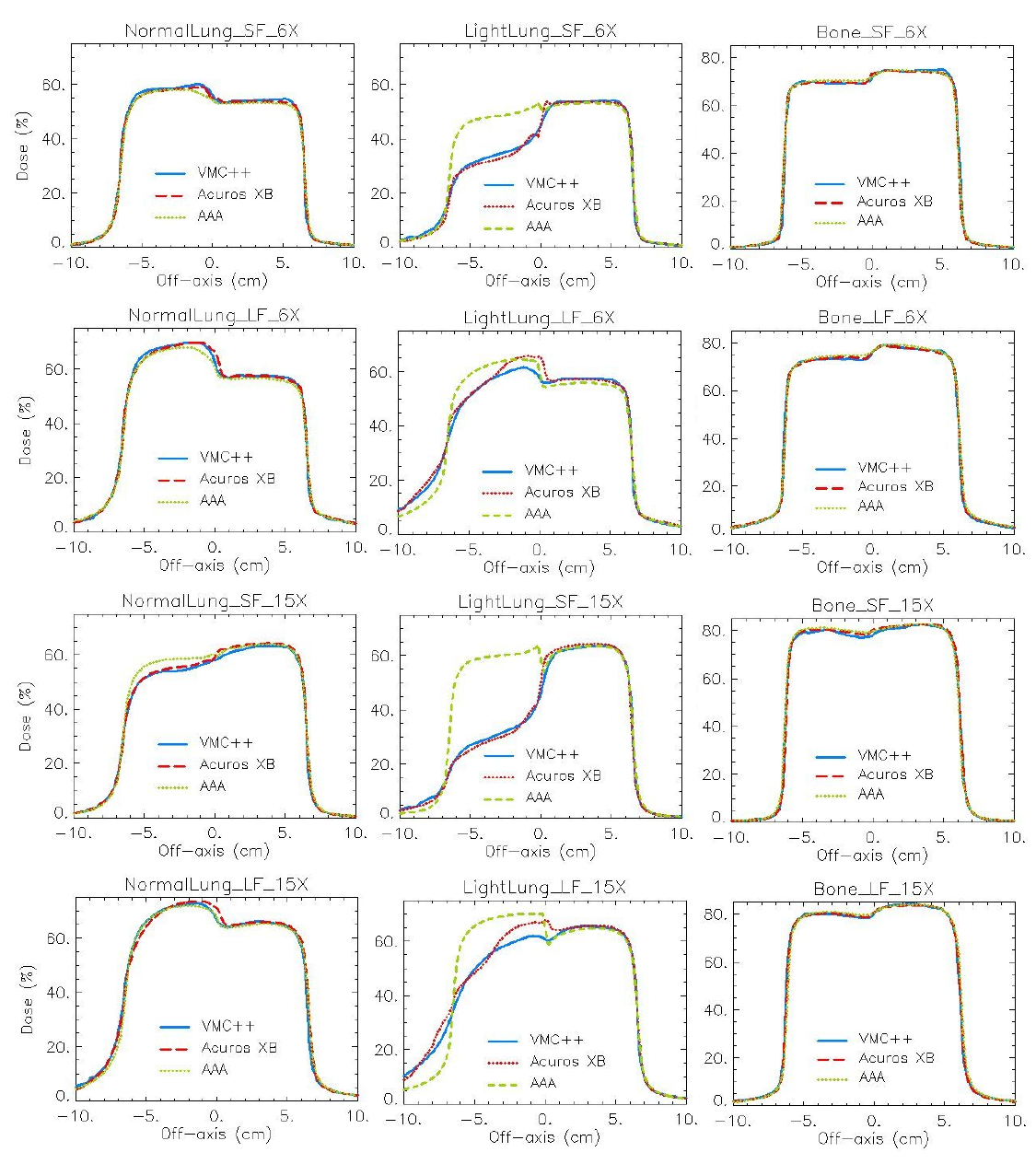

Supplement: Additional file 4 — Dose profiles rescaled to water for phantom A. Profiles at mid-depth of the heterogeneity insert. Dose rescaled to water calculations for VMC++, Acuros XB version 10, and AAA in phantom A. In columns: Normal Lung, Light Lung, Bone; in rows: SF and LF for 6X, SF and LF for 15X. [file 1748-717X-6-82-S4.PPT]

## Slide 1
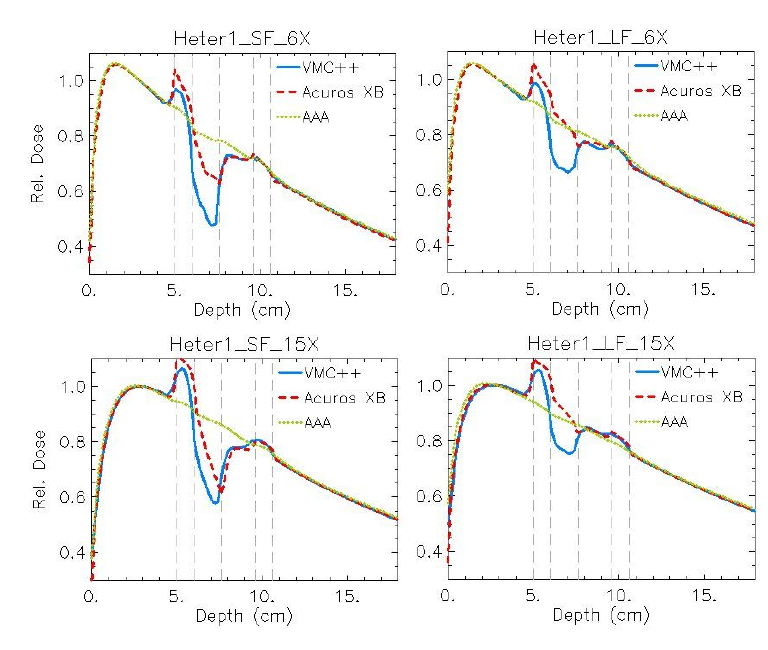

Supplement: Additional file 5 — DD to water for phantom B. Depth dose curves (DD) at beam central axis. Dose to water calculations for VMC++, Acuros XB version 10, and AAA in phantom B. In columns: SF, LF; in rows: 6X, 15X. [file 1748-717X-6-82-S5.PPT]

## Slide 1
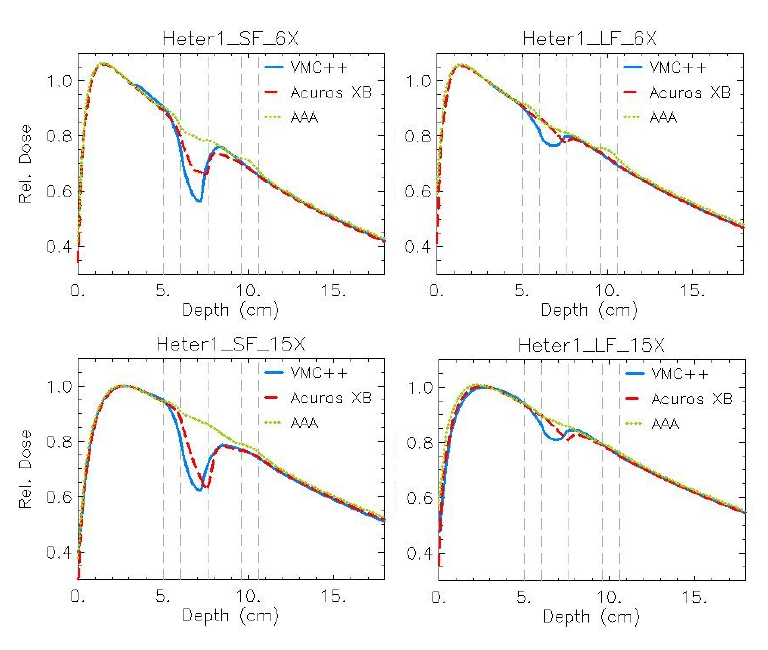

Supplement: Additional file 6 — DD rescaled to water for phantom B. Depth dose curves (DD) at beam central axis. Dose rescaled to water calculations for VMC++, Acuros XB version 10, and AAA in phantom B. In columns: SF, LF; in rows: 6X, 15X. [file 1748-717X-6-82-S6.PPT]

## Slide 1
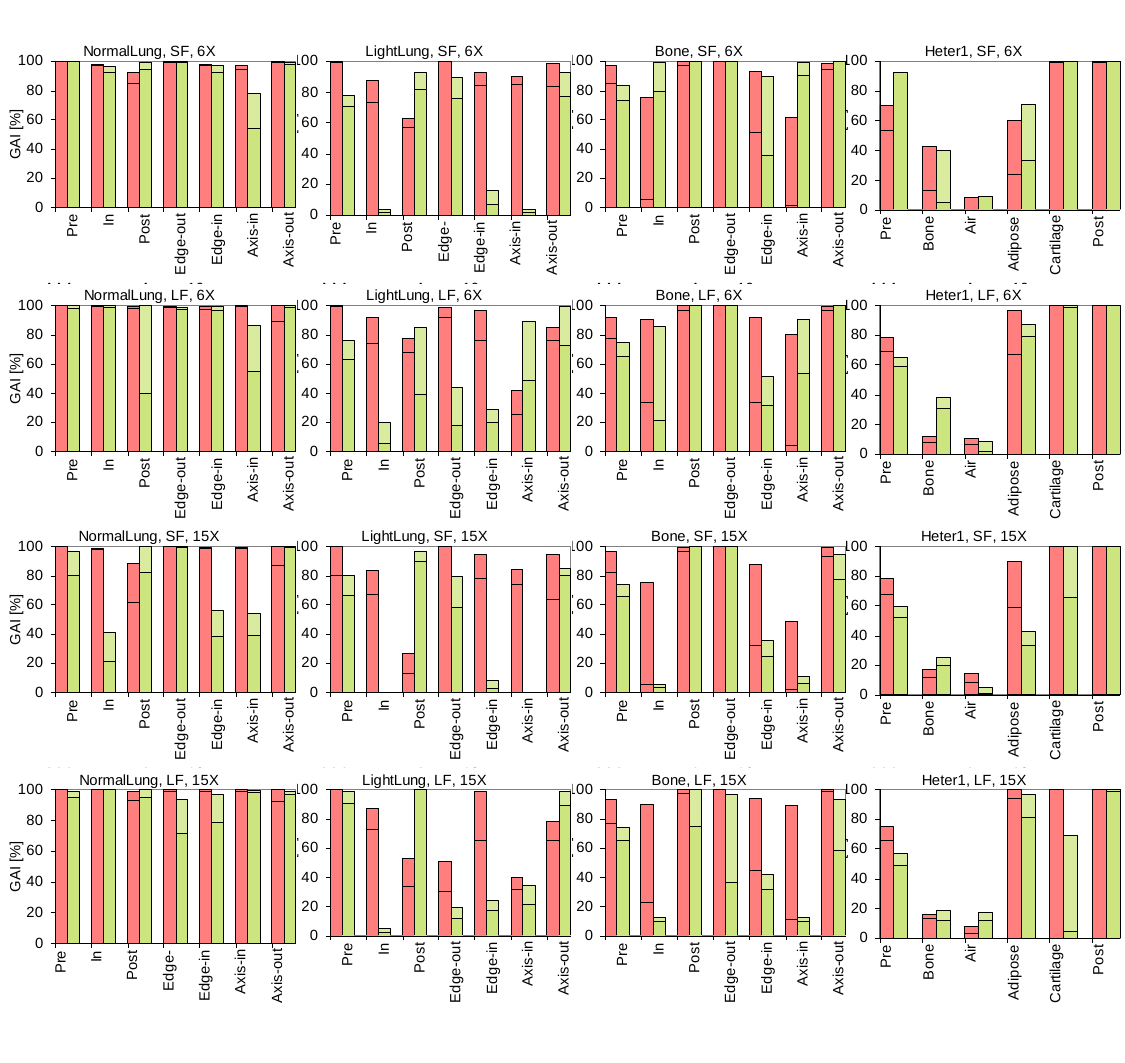

Supplement: Additional file 7 — GAI for dose to water. Histograms of the GAI. Global gamma calculation for each sector of phantoms A and B, for dose to water calculations for Acuros XB version 10 (red horizontal hatching) and AAA (green diagonal hatching). Each bin represents the two threshold results of 2%, 2 mm (thin cross-hatching) and 3%, 3 mm (thick cross-hatching). In columns: Normal Lung, Light Lung, Bone, phantom B; in rows: SF and LF for 6X, SF and LF for 15X. [file 1748-717X-6-82-S7.PPT]

## Slide 1
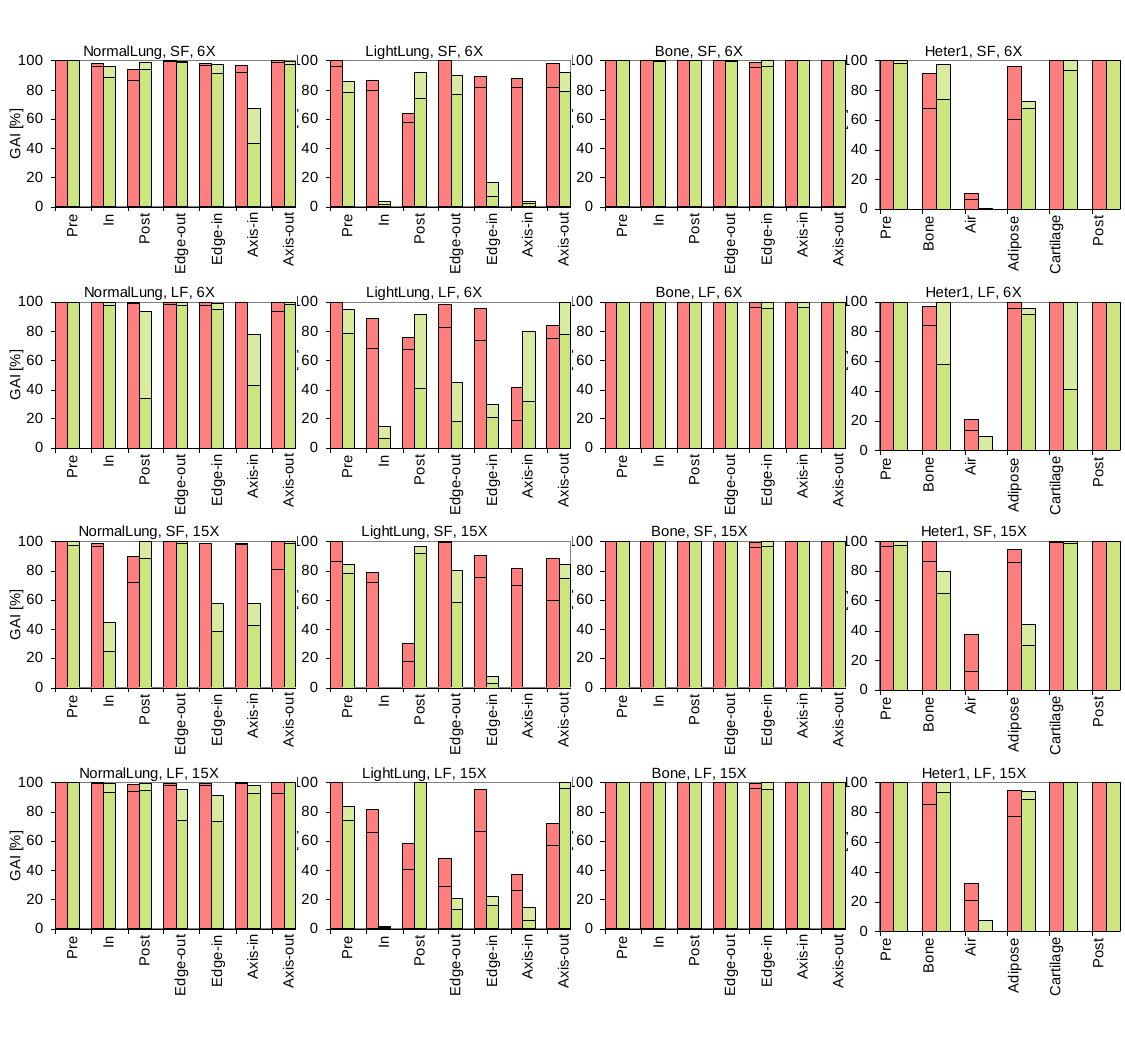

Supplement: Additional file 8 — GAI for dose rescaled to water. Histograms of the GAI. Global gamma calculation for each sector of phantoms A and B, for dose rescaled to water calculations for Acuros XB version 10 (red horizontal hatching) and AAA (green diagonal hatching). Each bin represents the two threshold results of 2%, 2 mm (thin cross-hatching) and 3%, 3 mm (thick cross-hatching). In columns: Normal Lung, Light Lung, Bone, phantom B; in rows: SF and LF for 6X, SF and LF for 15X. [file 1748-717X-6-82-S8.PPT]
